# SARS-CoV-2 Omicron Specific Mutations Affecting Infectivity, Fusogenicity, and Partial TMPRSS2-Independency

**DOI:** 10.3390/v15051129

**Published:** 2023-05-09

**Authors:** Romano Strobelt, Karin Broennimann, Julia Adler, Yosef Shaul

**Affiliations:** Department of Molecular Genetics, Weizmann Institute of Science, Rehovot 7610001, Israel

**Keywords:** omicron functional mutants, SARS-CoV-2 mutants, viral-mediated membrane fusion, lenti-Spike pseudovirus, TMPRSS2 in viral infection, SARS-CoV-2 endosomal infection

## Abstract

The COVID-19 pandemic resulted from the global spread of the severe acute respiratory syndrome coronavirus 2 (SARS-CoV-2). Since its first appearance in 2019, new SARS-CoV-2 variants of concern (VOCs) have emerged frequently, changing the infection’s dynamic. SARS-CoV-2 infects cells via two distinct entry routes; receptor-mediated endocytosis or membrane fusion, depending on the absence or presence of transmembrane serine protease 2 (TMPRSS2), respectively. In laboratory conditions, the Omicron SARS-CoV-2 strain inefficiently infects cells predominantly via endocytosis and is phenotypically characterized by decreased syncytia formation compared to the earlier Delta variant. Thus, it is important to characterize Omicron’s unique mutations and their phenotypic manifestations. Here, by utilizing SARS-CoV-2 pseudovirions, we report that the specific Omicron Spike F375 residue decreases infectivity, and its conversion to the Delta S375 sequence significantly increases Omicron infectivity. Further, we identified that residue Y655 decreases Omicron’s TMPRSS2 dependency and entry via membrane fusion. The Y655H, K764N, K856N and K969N Omicron revertant mutations, bearing the Delta variant sequence, increased the cytopathic effect of cell–cell fusion, suggesting these Omicron-specific residues reduced the severity of SARS-CoV-2. This study of the correlation of the mutational profile with the phenotypic outcome should sensitize our alertness towards emerging VOCs.

## 1. Introduction

Severe acute respiratory syndrome coronavirus 2 (SARS-CoV-2) is the causative agent for the ongoing coronavirus disease 19 (COVID-19) pandemic [1,2,3]. Since its first appearance in Wuhan, China, in 2019, SARS-CoV-2 has undergone several mutations [4,5]. In particular, variants of concern (VOCs), such as SARS-CoV-2 Delta (Delta), have displayed altered infection dynamics, possibly by escaping the immune response acquired against earlier SARS-CoV-2 variants, to spread more efficiently [5,6,7]. In November 2021, SARS-CoV-2 B.1.1.529 was first described in South Africa and was immediately announced as a new VOC named Omicron by the WHO [8,9]. A few weeks later, Omicron became the dominant strain in parts of Africa, Europe, and the USA [10,11,12]. Omicron and its subvariants remained the predominant variants worldwide for over a year [9,13].

Omicron contains around 50 mutations compared to the initial SARS-CoV-2 strain (Wuhan) with over 30 amino acid (aa) variations in the Spike, the viral surface glycoprotein [14,15]. Although most of the mutations are localized in the Spike’s receptor-binding domain (RBD), angiotensin-converting-enzyme (hACE2) remains Omicron’s cellular receptor with an even higher affinity [15,16,17,18]. SARS-CoV-2 interaction with hACE2 initiates cell entry via receptor-mediated endocytosis or membrane fusion, depending on the absence or presence of transmembrane serine protease 2 (TMPRSS2), respectively [19,20,21]. Omicron can infect cells in a TMPRSS2-independent manner, maximizing the receptor-mediated endocytosis route of infection [21,22,23,24]. The partial independence of TMPRSS2 might explain Omicron’s reduced fusogenic properties of syncytia formation in laboratory conditions. However, TMPRSS2-unrelated mutations can increase the Spike-mediated cell–cell fusion as well [23,25]. Therefore, identifying the Omicron-specific mutations that reduce infectiousness and pathogenicity should impact therapeutic strategies. In addition, correlating specific Spike residues with phenotypic outcomes might predict the features of the emerging VOC in advance.

In contrast to Omicron, VOC Delta predominantly enters cells via membrane fusion and is more infectious and fusogenic under laboratory conditions [23,24,25,26]. We created Spike chimeras by swapping between Omicron and Delta to pinpoint the functional Omicron mutations. We show that Omicron-specific F375 residue gives rise to inefficient infection and Y655 residue increases the endosomal path of infection. The latter increases infectivity but downregulates cell–cell fusion. The Omicrons’ unique K679, K856, and K969 residues also reduce cell–cell fusion, but are necessary for keeping Omicron’s maximal level of infection. These findings are expected to contribute to classifying the expected emergence of new SARS-CoV-2 variants based on their mutation profile.

## 2. Methods

### 2.1. Cell Culture

HEK293T, human embryonic kidney cells overexpressing SV40 large T-antigen (ATCC) and HEK293T-hACE2 were cultured in Dulbecco’s Modified Eagle’s Medium (DMEM, Gibco, Thermo Scientific, Waltham, MA, USA) containing 8% fetal bovine serum (Gibco) and 100 units/mL penicillin and 100 µg/mL streptomycin (Biological Industries, Beit Haemek, Israel). Additionally, HEK293T-hACE2 were constantly selected with 15 ng/mL blasticidin, as previously described [27]. HEK293T-hACE2-TMPRSS2 derived from HEK293T-hACE2 that were further transfected with pEFIRES-TMPRSS2-Flag plasmid and selected with 1.2 ng/mL puromycin one day before experiment. Cells were either harvested with Trypsin B solution (Biological Industries) or using a non-enzymatic protocol to harvest with phosphate-buffered saline (PBS) containing 1 mM EGTA before each experiment.

### 2.2. Plasmids

The pcDNA3.1-SARS2-Spike-Delta and -Omicron plasmids were obtained from Gideon Schreiber. We used overlapping primer (ST.1) for creating either point mutations or chimeras and subcloned them back in pcDNA3.1 using the closest restriction sites (RS) either NheI, KpnI, EcoNI, EcoRV or XhoI. List of used primers is attached in Supplementary Table S1 (ST.1). For chimera 215, we used RS EcoNI, for chimera 601 RS KpnI and for Omicron-Spike-F981L RS EcoRV.

### 2.3. Transfection, Generating of Pseudovirus, and Transduction

These methods were recently reported [27]. In brief, we used the calcium-phosphate (CaPO_4_) method to transfect cells in a 6 cm plate. The transfection mix was prepared by mixing 250 µL solution containing 8 µg DNA and 25 µL calcium chloride (CaCl_2_) with 250 µL two-times HEPES buffered saline (HBS2x) while vortexing. The transfection solution was immediately added to the 80% confluent cells. Eight h later, the medium was replaced with fresh growth medium.

For lenti-Spike preparation, HEK293T cells were transfected with pcDNA3.1-SARS2-Spike-Delta/Omicron (1.5 µg), pGIPZ-tGFP (3.5 µg) and pCMV-ΔR8.9 plasmids (3 µg). After 2.5 days, the lenti-Spike virion-containing medium was filtered through a 0.45 µm membrane filter (Sartorius, Beit Haemek, Israel). The medium with the virions was adjusted to equal amounts based on the RNA levels and added directly to cells and washed away after eight h. Hoechst 33342 solution (1:2000, Molecular Probes, Life Technologies, Carlsbad, CA, USA) was added after 1.5 days, before microscopically analyzing infection efficiency. The ratio of Hoechst-stained nuclei (all cells) and GFP-emitting cells (infected cells) was quantified by using the previously described ImageJ macro [27].

### 2.4. Cell-Fusion Assay

HEK293T cells were transfected with the respective Spike mutant, Fos-YFPc and RFP plasmid. The HEK293T-hACE2 were transfected with TMPRSS2, Jun-YFPn and RFP plasmids. RFP signal served as transfection control. After 1.5 days, the two sets of transfectants were non-enzymatically harvested and equal amounts of cells were mixed and plated in 1:1 ratio in a 24-well plate. For monitoring time of fusion, the 24-well plate was transferred to IncuCyte system (Sartorius), and images were taken in brightfield, GFP and RFP channel at half-an-hour intervals. Data analyses were carried out with IncuCyte integrated software.

### 2.5. Graphs and Statistics

Graphs and statistics were produced using GraphPad Prism software. Error bars represent standard error of the mean and *t*-tests are always two-tailed. Shapiro–Wilk test confirmed normal distribution of our data. If not specified otherwise, the calculations are based on three biological replicates.

## 3. Results

### 3.1. The Omicron F375 Residue Decreases Infection

**Figure 1:**
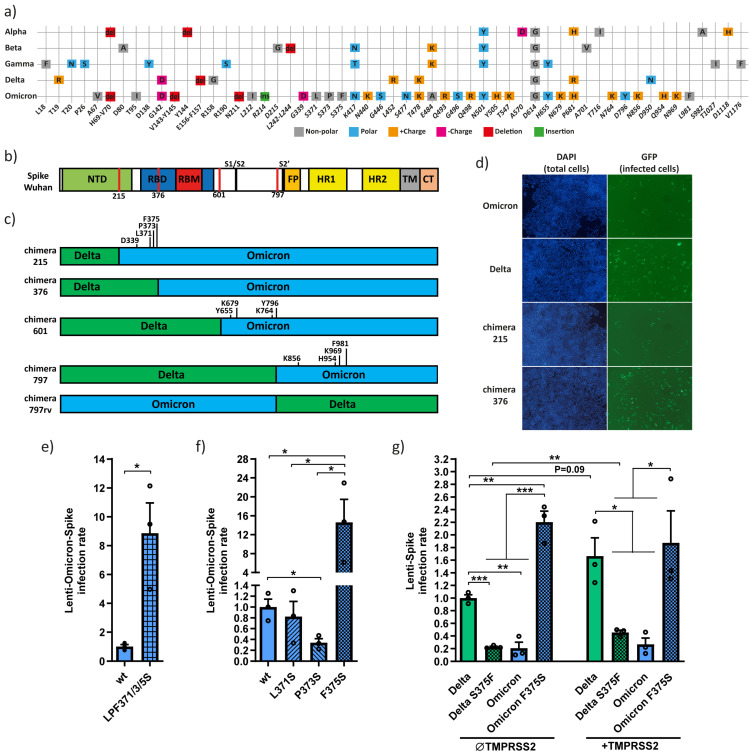
Omicron-Spike F375 residue downregulates Omicron pseudovirus infection. (**a**) Comparison of Omicron Spike with previous SARS-CoV-2 VOCs. While earlier VOCs had fewer than 10 mutations within the Spike compared to Wuhan, Omicron possesses 34 mutations. (**b**) The Spike protein of Wuhan is composed of 1273 aa and several functional regions. These include the N-terminal domain (NTD) (13–305 region) and the receptor binding domain (RBD) (319–541 region). The RBD 437–508 region directly interacts with hACE2. Spike can be cleaved by furin or cathepsin-L at R685 (S1/S2) or/and by TMPRSS2 at R815 (S2’). The fusion peptide (FP) is located at 816–855 region. Both heptad repeats (HR1 and HR2) are located at 912–984 and 1163–1213, respectively. The C-terminus of the Spike contains the transmembrane (TM) and cytosolic tail (CT). The functional regions differ between VOCs due to minimal deletion or insertion. Red lines describe the breakpoint between Delta and Omicron chimeras. (**c**) Schematic description of the constructed Delta-Omicron Spike chimeras. Some of the Omicron unique mutations are highlighted. (**d**) Lenti-Spike with chimera 376 is more infectious compared to chimera 215. Lentiviruses were generated with the indicated Spike constructs. Equal amounts of virions, as was determined based on RNA levels, were used to transduce the HEK293T-hACE2-TMPRSS2 cell line for 8 h. Cells were treated with Hoechst after 1.5 days, and microscopic images were taken. The images are representative of three independent replicates. (**e**) The Omicron triple mutant; LPF371/3/5S increases lenti-Spike infection rate. Lentivirus expressing either wt or LPF371/3/5S Omicron Spike residues were prepared and used to transduce HEK293T-hACE2. The infection rate was calculated after 1.5 days shortly after Hoechst staining. Infection rate was calculated by normalizing to wt Omicron. (**f**) Omicron Spike F375S mutant increases infectivity. Omicron Spike was either mutated at L371S, P373S or F375S, and the generated SARS-CoV-2 pseudoviruses were used to transduce HEK293T-hACE2 cells as described above. (**g**) Delta Spike S375F mutant reduces lenti-Delta-Spike infection rate. HEK293T-hACE2 cells were either transfected with pEFIRES alone or together with TMPRSS2 plasmids and treated with 1.2 µM puromycin after 1.5 days. Cells were transduced with lenti-Spike Omicron, Delta or respective mutant as described above. Infection rate was calculated by normalizing to Delta in TMPRSS2-negative cells. Student-*t*-test was used for measuring statistical significance in panels (**e**–**g**). * = *p* ≤ 0.05; ** = *p* ≤ 0.01; *** = *p* ≤ 0.001.

The abundance of mutations within the Spike protein of Omicron exceeds all previous VOCs (Figure 1a,b) [14,15]. The Omicron Spike’s mutational burden downregulates infectivity and fusogenicity [6,7,23,25] and yet is widely spread. We generated Delta–Omicron chimera constructs to investigate the role of the unique Omicron mutations (Figure 1c). To avoid the generation of new gain-of-function SARS-CoV-2 variants, we used the surrogate SARS-CoV-2 pseudovirus (lenti-Spike)-expressing GFP to follow infection. We transduced HEK293T cells expressing the viral receptor HEK293T-hACE2, as previously described [27]. We constructed the 215 and 376 chimeras, where the equivalent Delta Spike regions replaced the 215 or 376 N-terminal regions of the Omicron Spike (Figure 1c). Increased lenti-Spike infection was observed with the 376-Delta–Omicron Spike chimera, compared to both Omicron and 215-Delta–Omicron chimera (Figure 1d). Within the 215–376 Spike region, there were three non-polar Omicron unique residues in close proximity: L371, P373 and F375. To examine the importance of these residues, we constructed an Omicron-Spike-LPF371/3/5S mutant bearing the equivalent Delta sequence (L371S, P373S and F375S). Omicron-Spike-LPF371/3/5S pseudovirus was about eight times more infectious (Figure 1e), suggesting that these residues downregulate Omicron’s infectivity. Afterwards, we mutagenized these residues one by one and found that the F375S mutation increased the infection rate (Figure 1f). The Omicron F375 residue, therefore, might be responsible for Omicron decreased infectivity. To verify this point, we conducted a reciprocal experiment by generating a Delta Spike S375F mutant. This Delta mutant inefficiently infected cells (Figure 1g). Thus, the unique Omicron Spike F375 residue might be the reason for the low Omicron infectivity in tissue culture.

Next, we investigated TMPRSS2 dependency in infection and found that TMPRSS2 did not improve the infection of wt Omicron or the Omicron Spike F375S mutant. However, TMPRSS2 increased Delta and Delta S375F infection rates. Thus, the Omicron F375 unique residue impacts infection regardless of TMPRSS2.

### 3.2. Omicron Y655 Residue Supports Endosomal Route of Infection

**Figure 2:**
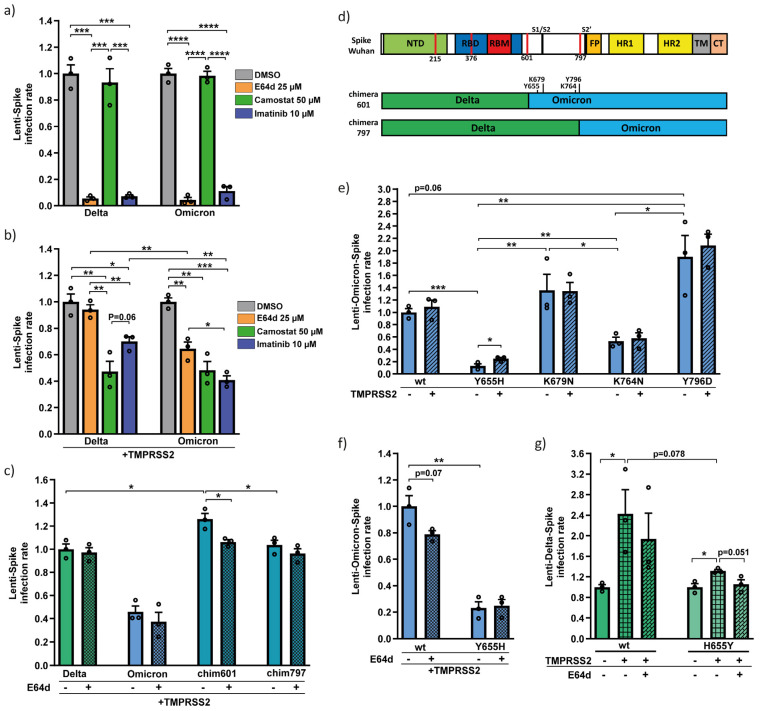
The Omicron Y655 residue increases infection rate and reduces TMPRSS2 dependency. (**a**) SARS-CoV-2 entry inhibitors have similar effects against lenti-Delta- and lenti-Omicron-Spike in TMPRSS2-negative cells. HEK293T-hACE2 cells were treated with each; DMSO (untreated), E64d, camostat and imatinib, 2 h before infection with either lenti-Delta- or lenti-Omicron-Spike and further treated as described above. Infection rate was calculated by normalizing to DMSO treated cells. (**b**) Lenti-Omicron-Spike but not Lenti-Delta-Spike infection was inhibited by E64d in TMPRSS2-positive cells. HEK293T-hACE2 cells were transfected with pEFIRES-TMPRSS2 and treated with 1.2 µM puromycin 1.5 d later. Next day, cells were treated as above. (**c**) Lenti-Spike chimera 797 was not inhibited by E64d in TMPRSS2-positive cells. The indicated different lenti-Spike virions were used to transfect HEK293T-hACE2-TMPRSS2. Cells were treated with E64d and infected with lenti-Spike as described above. Infection rate was calculated by normalizing to untreated Delta. (**d**) Scheme of the Delta-Omicron-Spike chimeras compared to functional regions of Wuhan Spike with Omicron unique residues highlighted. (**e**) Lenti-Omicron-Spike Y655H infectivity increases in TMPRSS2-positive cells in comparison to TMPRSS2-negative cells. HEK293T-hACE2 cells were transfected with either pEFIRES or pEFIRES-TMPRSS2 and puromycin treated as above. Infection rate was calculated by normalizing to wt Omicron. (**f**) E64d treatment is ineffective against lenti-Omicron-Y655H-Spike infection. HEK293T-hACE2-TMPRSS2 cells were generated and treated as described above. (**g**) Delta H655Y mutant was poorly TMPRSS2 dependent. Cells were treated as described above, and infection rate was calculated by normalizing to untreated TMPRSS2-negative cells. Data were statistically analyzed by student-*t*-test: * = *p* ≤ 0.05; ** = *p* ≤ 0.01; *** = *p* ≤ 0.001; **** = *p* ≤ 0.0001.

To study the Omicron route of infection, we treated cells with specific inhibitors. E64d inhibits cathepsin-L, an essential enzyme in regulating the endosomal fusion [20,28]; whereas, camostat inhibits TMPRSS2 serine protease, an essential enzyme in regulating the membrane route [20]. Imatinib inhibits both pathways by directly binding to Spike [27,29]. In TMPRSS2-negative cells, infection of both lenti-Delta and lenti-Omicron Spike was inhibited by E64d and imatinib, but not by camostat (Figure 2a), verifying their endosomal route. In TMPRSS2-positive cells, the membrane fusion is expected to be the major entry route. Under this condition, E64d inhibited the lenti-Omicron-Spike but not lenti-Delta-Spike infection, while camostat inhibited both. Thus, in the presence of TMPRSS2, Delta exclusively infected via membrane fusion, whereas Omicron also infected cells endosomally (Figure 2b). Imatinib inhibited the two Spike variants but was generally more effective against the lenti-Omicron Spike.

Having demonstrated that, unlike the Delta Spike, Omicron endosomally infects TMPRSS2 positive cells, we next aimed to identify the involved unique Omicron residues. HEK293T-hACE2-TMPRSS2 cells were infected with lenti-Delta, -Omicron and their chimera constructs and treated with E64d before and throughout infection. E64d decreased the infection of 601 but not the 797 chimera Spike, indicating the latter did not infect endosomally (Figure 2c). Four of the Omicron unique residues, Y655, K679, K764 and Y796, are found in the sequence of the 601 but not the 797 chimeras (Figure 2d). To investigate the role of these residues one by one in the context of Omicron, we generated Omicron Spike mutants bearing the equivalent Delta Spike sequence; Y655H, K679N, K764N and Y796D, and infected TMPRSS2-negative and -positive HEK293T-hACE2 cells. Interestingly, Omicron Y655H and K764N mutations markedly reduced infectivity compared to the wt Omicron (Figure 2e), suggesting that in the context of Omicron, residues Y665 and K764 are required for efficient infection. Infection of Omicron Y655H mutant was TMPRSS2 responsive (Figure 2e) and E64d irresponsive (Figure 2f). Thus, in the context of the Delta variant, the histidine residue at position 655 supports the TMPRSS2-dependent membrane route of infection. Next, we conducted a reciprocal experiment and generated a Delta H655Y mutant and found it to be poorly TMPRSS2 responsive (Figure 2g). These data suggest the Spike Y655 residue is responsible for Omicron’s partial TMPRSS2 independence and for an increased endosomal route.

### 3.3. Residues Y655, K764, K856, and K969 Attenuate Omicron-Spike Membrane Fusion

**Figure 3:**
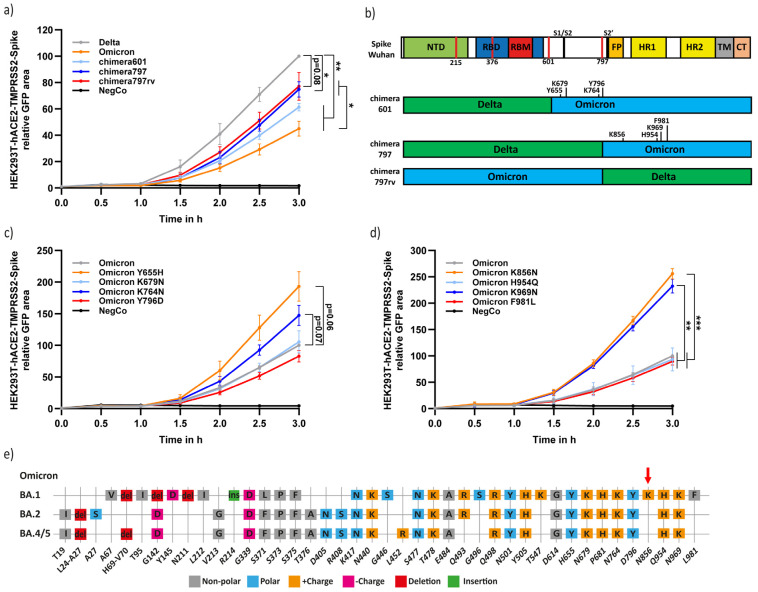
Omicron Spike Y655, K764, K856 and K969 unique residues decrease cell–cell fusion and syncytia formation. (**a**) Delta-Spike has the highest fusion efficiency compared to the different Delta–Omicron Spike chimera. HEK293T cells were transfected with Spike chimeras and Fos-YFP_c,_ and HEK293T-hACE2 cells were transfected with TMPRSS2 and Jun-YFP_n_. Cells were mixed in a 1:1 ratio after 1.5 days, and images were taken with the IncuCyte system at half-hour intervals and analyzed with IncuCyte Software. The total GFP area (µm^2^/Image) was adjusted to ascertain individual confluency. The obtained data from four biological replicates were normalized to that of 3 h Delta results, and the area under the curve was calculated. Results summarize four biological replicates. (**b**) Scheme of the constructed Delta-Omicron-Spike chimeras compared to functional regions of Wuhan Spike. The indicated point mutations highlight the differences between Spike chimera 601, 797, and 797rv. (**c**) The Y655H and K764N mutations increased Omicron fusogenicity. HEK293T and HEK293T-hACE2 cells were treated and analyzed as described above. Data were normalized to wt Omicron and represent three biological replicates. (**d**) Omicron Spike K856N and K969N mutations increased cell fusion. The experiment was performed and analyzed as above. (**e**) Schemes of Omicron subvariants. The sequences of the new Omicron variants showed higher similarity than the first Omicron strain (BA.1). The unique residues are located mainly in NTD and RBD/RBD domains. K856N increases fusion efficiency in all new Omicron subvariants highlighted with a red arrow. For statistical analysis, we used student-*t*-test. * = *p* ≤ 0.05; ** = *p* ≤ 0.01; *** = *p* ≤ 0.001.

Next, we compared the different variants for their rate of cell–cell fusion and syncytia formation. To this end, we employed our recently described Spike-mediated cell–cell fusion assay using a split YFP approach [27]. We transfected HEK293T with the Spike chimeras and Fos-YFP_c_ and mixed them after 1.5 d with the HEK293T cells coexpressing hACE2, TMPRSS2 and Jun-YFP_n,_ in a 1:1 ratio. Upon fusion of these two cells, the split YFP was complemented with Fos and Jun heterodimerization [30,31]. The Delta-Spike was the most effective in mediating cell–cell fusion (Figure 3a). The fusion level of the Delta 797-Omicron Spike chimera and the Omicron 797-Delta chimera (797rv) was slightly higher than both the Delta Spike 601-Omicron chimera and wt Omicron-Spike (Figure 3a), suggesting that the Omicron-Spike region 601 to 797 and also the Spike 797 upstream region each decrease fusion.

Within the Spike 601–797 region, there are four unique Omicron residues; Y665, K679, K764, and Y796 (Figure 3b). To identify which of these residues regulates fusion, we mutagenized the residues one by one to bear the equivalent Delta sequence; Y655H, K679N, K764N and Y796D, and found that Y655H and K764N mutations increased fusion and syncytia formation (Figure 3c). These data suggest that, in the context of Omicron, the Y655 and K764 residues downregulate fusion.

The Omicron 797-Delta chimera (797rv), lacking these residues, also increased fusion compared to naïve Omicron and chimera 601. We, assumed, therefore, that residues upstream of the Omicron 797 downregulate fusion as well. There are four unique Omicron residues within this region, K856, H954, K969 and F981; each was mutated to bear the Delta equivalent sequence (K856N, H954Q, K969N and F981L). We revealed that K856N and K969N mutations increased fusion rate and, therefore, concluded that the Omicron K856 and K969 residues downregulate fusion (Figure 3d). Notably, in agreement with our findings, the Omicron subvariants BA.2, BA.4, and BA.5 are active in syncytia formation and bear the Delta N856 residue (Figure 3e) [32,33].

### 3.4. Functional Analysis of the Different Unique Omicron Spike Residues in Combination

**Figure 4:**
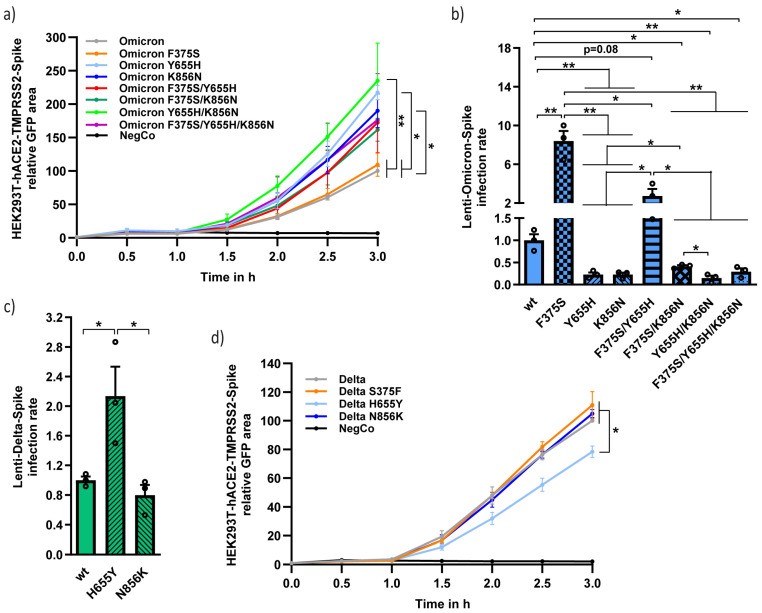
Analysis of the different combinations of functional unique Omicron Spike residues. (**a**) The Y655H and N856K Omicron-Spike mutants, each and in combination increased fusogenicity. HEK293T cells were transfected as above to monitor fusion rate. (**b**) Omicron-Spike-K856N mutant reduced infection rate. Lenti-Omicron-Spike mutants were created and used to transfect HEK293T-hACE2 cells as described above. Infection rate was calculated by normalizing to wt Omicron. (**c**) Delta-Spike H655Y but not N856K mutant increased infectivity. The indicated lenti-Delta-Spike constructs were used to infect HEK293T-hACE2 as described above. Infection rate was calculated by normalizing to wt Delta. (**d**) The Delta H655Y mutant decreased Delta-Spike fusion rate. Experiments were conducted as described above, and the data were normalized by Delta values. Statistics were carried out with student-*t*-test. * = *p* ≤ 0.05; ** = *p* ≤ 0.01.

Next, we investigated the phenotypic behavior of the unique and functional Omicron Spike residues in combination. Altogether, we have shown that Omicron Spike residue F375 reduces infection, Y655 decreases TMPRSS2 responsiveness, and K856 decreases cell–cell fusion rate. We prepared Omicron-Spike mutants, where these three residues were mutated to bear the Delta equivalent sequences (F375S, Y655H, and K856N) in different combinations, and examined their phenotypic manifestations. The F375S mutation did not increase the fusion rate. The F375S mutation, however, reduced the fusion rate of Y655H and K856N double mutants, but not of each single mutant (Figure 4a). The Omicron Spike Y655H/K856N double mutant was the most fusogenic, suggesting that it is active synergistically to elicit syncytia. Interestingly, the lowest infection rate was obtained with the Omicron Y655H/K856N double mutant and F375S mutant alone or in combination with Y655H, but not with K856N, improving infection (Figure 4b).

Since the Omicron Y655H and K856N mutants, bearing the Delta equivalent sequence, were poorly infectious, we assumed that the Omicron Y655 and K856 residues improved infection. To test this hypothesis, we mutagenized the Delta Spike to bear the Omicron equivalent residues to construct the H655Y and N856K mutants. Only Delta Spike H655Y was revealed to increase infection (Figure 4c). Furthermore, H655Y mutation, but not N856K, decreased Delta-Spike-mediated fusion (Figure 4d). Thus, a single point mutation in Delta Spike (H655Y) was sufficient to increase infectivity and to decrease fusogenicity.

## 4. Discussion

New mutations of SARS-CoV-2 bear the risk of escaping immune tolerance, increasing infectivity, and enhancing pathogenicity [5,34]. To evaluate the risk of future VOCs, it is crucial to understand the mutations of previous VOCs and their phenotypic and pathogenic impact. This study attempts to summarize the key mutations that led to phenotypical changes in Omicron infection, namely infectivity, fusogenicity, and the route of cell entry.

Unexpectedly, Omicron, despite being rapidly spread, is poorly infectious in laboratory conditions compared to VOC Delta [23,25,26]. We identified the unique F375 residue as one of the main residues regulating Omicron infectivity. We showed that by mutating F375 to bear the Delta variant equivalent sequence, F375S increases infectivity (Figure 1f,e). Close to F375 are the Omicron L371 and P373 unique residues. All three residues change the Delta polar serine to non-polar residues (Figure 1a). Interestingly, this region shapes a binding pocket for linoleic acid that changes between an open and a closed Spike conformation [35,36]. Adding hydrophobic residues at this region may prevent fatty acid binding and keep the Spike in an open conformation, potentiating SARS-CoV-2 in evading the immune response [15]. The immune evasion is expected to compensate for the decrease in Omicron infectivity. Omicron new subvariants, such as BA.2, BA.4, and BA.5, all keep the same genetic traits toward non-polar residues (T376A) [14,32,33]. We predict that mutations leading to an open Spike configuration will be more prevalent in the future.

The cellular protease TMPRSS2 directs SARS-CoV2 infection exclusively to membrane fusion [21,37]. However, the Omicron pseudovirus endosomally infects cells even in the presence of TMPRSS2 (Figure 2b) and TMPRSS2 ectopic expression does not improve infectivity (Figure 2d) [23,25]. In agreement with Hu et al. and Yamamoto et al., we showed that the Y655 residue determines Omicron’s preference for endosomal entry [38,39]. In addition, we show here that the Y655 residue is essential for Omicron to retain maximal infectivity (Figure 2e,f). Interestingly, the Delta H655Y mutant, bearing the equivalent Omicron Y655 residue, acquires some of the Omicron Spike features in improving the endosomal entry route even in the presence of TMPRSS2 (Figure 3g), being more infectious (Figure 4c), and at the same time less active in cell–cell fusion and syncytia formation (Figure 4d).

Omicron is less infectious than Delta in laboratory conditions yet spreads rapidly [10,23,24,25]. The reason is probably due to Omicron’s partial TMPRSS2 independency, resulting in Omicron’s broader cellular target, such as the upper respiratory tract cells [23,40,41,42]. Therefore, the Spike Y655 residue that allows the virus to infect endosomally, might expand cell tropism by sensitizing the TMPRSS2 negative cells. This possibility needs to be further verified by mutating the Spike of other VOCs to bear the Y655 residue and infecting upper and lower respiratory tract cells.

We demonstrated that Omicron-Spike K796N, K856N, and K969N mutations showed improved cell fusion (Figure 3b,c). Therefore, these three K residues might antagonize fusion. Interestingly, Omicron variants BA.2, BA.4, and BA.5 bearing N856 not K856 residue are more fusogenic than BA.1 variant [32,33]. These data may suggest that K856 is sufficient to downregulate fusion. However, Delta-Spike N856K mutant remained as fusogenic as wt Delta, meaning that in the context of Omicron, the effect of the K856 residue is dependent on some other Spike residues. In any case, although the Omicron K856 residue decreased cell–cell fusion, it was necessary for retaining the Omicron maximal infection rate (K856N, Figure 4b), possibly via the endosomal infection route.

**Figure 5:**
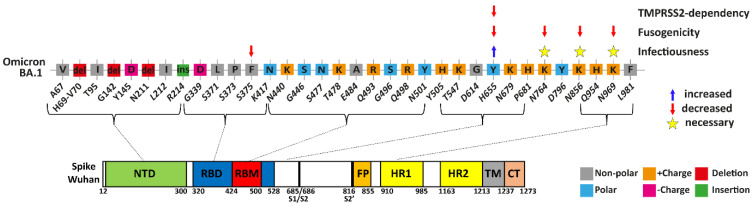
Summary of Spike mutations affecting Omicron infectivity, fusogenicity and TMPRSS2-independency. Highlighted are the Omicron residues that differ from wt SARS-CoV-2, and their location within the functional regions of the Spike. F375 Spike residue led to a significant reduction in Omicron infectivity, while Y655 residue seems to have a slight increase in infectivity. All Spike K764, K856, and K969 residues negatively affect fusogenicity, but are necessary for keeping the maximal infection rate. Y655 residue decreases TMPRSS2-dependency and allow virions to enter TMPRSS2-positive cells through endosomal fusion.

The same Spike residues that are linked with decreased fusogenicity, namely Y655, K796, K856, and K969, seem also to be necessary for Omicron infection (Figure 5). New Omicron variants bearing one or more of the equivalent Delta sequence are expected to increase pathogenicity. However, since these residues are also expected to reduce infection, they are evolutionarily inferior. Furthermore, a significant increase in infectivity through the acquirement of the F375S mutation is not expected in future Omicron sublineages unless they bear the K856 residue as well (Figure 4b).

In this study, we used mainly SARS-CoV-2 surrogate pseudovirus, an established method to study SARS-CoV-2 entry [20,27,43,44]. However, the pseudovirus does not recapitulate the complete SARS-CoV-2 life cycle by producing progeny for further spreading [19,45]. In addition, SARS-CoV-2 non-structural proteins that overcome the cellular innate immune response are also missing [46]. An additional limitation may be the construct’s expression levels and the resulting Spike protein coating on the virus particles that could differ between constructs and experiments [43,44]. To overcome this constraint, we quantified RNA and tested the proteins expression (Figure S1).

Omicron and the emerging subvariants are less severe than Delta but are more prevalent. Due to the high number of infected individuals, the total number of hospitalized COVID-19 patients was higher for Omicron- than for Delta-infected patients [47,48,49,50]. Therefore, new variants with increased infectiousness or severity bear the risk of even higher hospitalization and have to be prevented for the sake of the healthcare system. This study aimed to identify the key amino-acid residues determining the infectivity and pathogenicity of SARS-CoV-2 and the emerging variants. The findings should raise awareness in evaluating the potential risk of new VOCs.

## Data Availability

Not applicable.

## Supplementary Materials

The following supporting information can be downloaded at: https://www.mdpi.com/article/10.3390/v15051129/s1, Figure S1: Spike protein expression fluctuates around the same mean HEK293T were transfected with respective Spike construct, and cells were harvested for western blot analysis after 1.5d. Band intensities were calculated with ImageJ, and values were adjusted to the actin level. Values were normalized to the Omicron band. Bar graph shows measurements of three biological replicates; Table S1: List of Primer used for cloning of described constructs.
