# Supplementary material for: SARS-CoV-2 Omicron Specific Mutations Affecting Infectivity, Fusogenicity, and Partial TMPRSS2-Independency
Source: Viruses. 2023 May 9;15(5):1129. doi: 10.3390/v15051129 (PMC10223509; doi:10.3390/v15051129)
Supplement: Supplementary file 1 [file viruses-15-01129-s001.zip › viruses-2349363-supplementary.pdf]

Supplement

SARS-CoV-2 Omicron specific mutations affecting infectiousness, fusogenicity, and partial TMPRSS2-independency

Romano Strobelt, Karin Broennimann, Julia Adler, Yosef Shaul*

Department of Molecular Genetics, Weizmann Institute of Science, Rehovot, Israel
* Corresponding author

Name	Sequence
Spike_Fw_NheI_pcDNA3.1	aagctggctagcatgttcgtgttcct
Spike-Flag_Rv_XhoI_pcDNA3.1	ATGCCCctcgagTCActtgtcgtcatcgtctttgtagtctgtatagtgcagtttgacgcccttc
chimera376_Fw_Delta-Omicron	ttcaaatgttatggtgtttcgccaacaaagctgaa
chimera376_Rv_Delta-Omicron	ttcagctttgttggcgaaacaccataacatttgaa
chimera797_Fw_Delta-Omicron	ttcggcggcttcaatttcagccaaatactcccaga
chimera797_Rv_Delta-Omicron	tctgggagtatttggctgaaattgaagccgccgaa
Delta-S371-S373-S375_Fw	agcgtgctgtacaatagcgccagcttcagcaccttcaaatgtta
Delta-S371-S373-S375_Rv	taacatttgaaggtgctgaagctggcgctattgtacagcacgct
Delta-S371_Fw	gtgctgtacaatAGCgccCCTttcTTc
Delta-S371_Rv	gAAgaaAGGgcGCTattgtacagcac
Delta-S373_Fw	tacaatCTGgccAGCttcTTcaccttc
Delta-S373_Rv	gaaggtgAAgaaGCTggcCAGattgta
Delta-S375_Fw	CTGgccCCTttcAGcaccttcaaatg
Delta-S375_Rv	catttgaaggtgCTgaaAGGggcCAG
Delta-H655_Fw	atcggcgccgagcacgtgaataata
Delta-H655_Rv	tattattcacgtgctcggcgccgat
Delta-N679_Fw	gacccagaccaaTagccACAgaagG
Delta-N679_Rv	CcttcTGTggctAttggtctgggtc
Delta-N764_Fw	cacccagctgaaTagagccctgacc
Delta-N764_Rv	ggtcagggctctAttcagctgggtg
Delta-D796_Fw	ccgccgatcaagGacttcggcggct
Delta-D796_Rv	agccgccgaagtCcttgatcggcgg
Delta-N856_Fw	ccagaagtttaacggtttgacagta
Delta-N856_Rv	tactgtcaaaccgttaaacttctgg
Delta-Q954_Fw	cgtggtgaatcagaatgcccaggccc
Delta-Q954_Rv	gggcctgggcattctgattcaccacg
Delta-N969_Fw	gctgagcagcaatttcggcgccatc
Delta-N969_Rv	gatggcgccgaaattgctgctcagc
Omicron-S375F_Fw	agcgccagcttcttcaccttcaaatg
Omicron-S375F_Rv	catttgaaggtgaagaagctggcgct
Omicron-H655Y_Fw	atcggcgccgagTacgtgaataatagc
Omicron-H655Y_Rv	gctattattcacgtActcggcgccgat
Omicron-N856K_Fw	gcccagaagtttaaAggtttgacagta
Omicron-N856K_Rv	tactgtcaaaccTttaaacttctgggc

Supplement Table S1: List of Primer used for cloning of described constructs

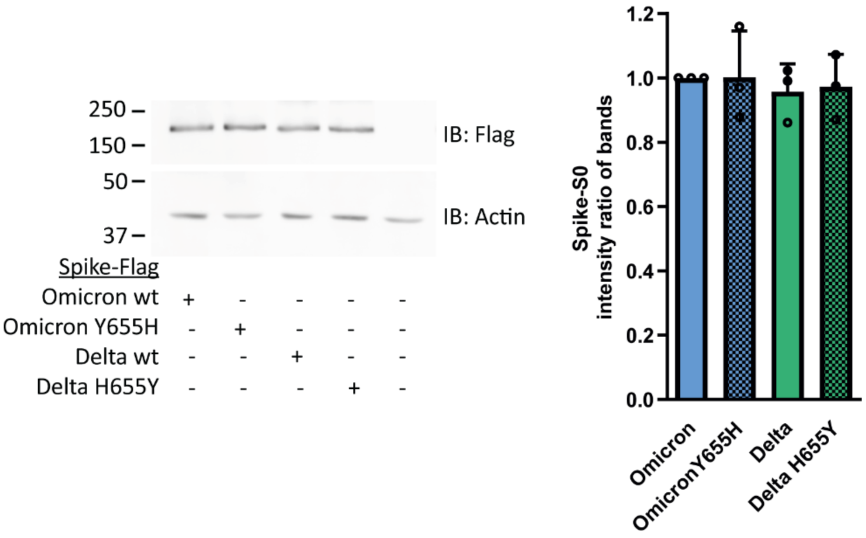

Figure S1: Spike protein expression fluctuates around the same mean HEK293T were transfected with respective Spike construct, and cells were harvested for western blot analysis after 1.5d. Band intensities were calculated with ImageJ, and values were adjusted to the individual actin band of each lane. Afterward, values were normalized to the Omicron band. Bar graph shows measurements of three repeats.
